# Real-World Effectiveness and Safety of Adding Trimetazidine to Standard Antianginal Therapy in Patients with Stable Angina: Egyptian Cohort Findings of the COMBINE Angina Study

**DOI:** 10.3390/medsci14060620

**Published:** 2026-09-30

**Authors:** Mohamed Sobhy, Mohamed Fahmy Elnoamany, Mohamed Loutfi, Sharaf Eldeen Mahmoud, Mohamed Abdel Ghany, Mohamed Kamal Salama, Adham Ahmed Abdeltawab, Yasser A. Abdelhady

**Affiliations:** 1 Faculty of Medicine, Alexandria University, Alexandria 21526, Egypt; drmloutfi@gmail.com; 2 Faculty of Medicine, Menoufia University, Shebin El-Kom 32511, Egypt; mnoamany@hotmail.com; 3 Faculty of Medicine, Sohag University, Sohag 82524, Egypt; sharaf003@gmail.com; 4 Faculty of Medicine, Assiut University, Assiut 71515, Egypt; m_koriem2004@yahoo.com; 5 Faculty of Medicine, Kafr Elsheikh University, Kafr Elsheikh 33516, Egypt; kfs_mss@yahoo.com; 6 Faculty of Medicine, Ain Shams University, Cairo 11566, Egypt; adham_abdeltawab@med.asu.edu.eg; 7 Faculty of Medicine, Beni Suef University, Beni Suef 62511, Egypt; yasser.abdelhady@icloud.com

**Keywords:** chronic coronary syndrome, stable angina, CCS classification, Seattle Angina Questionnaire

## Abstract

**Background/Objectives:** Stable angina is a significant health issue in Egypt, often showing poor responses to standard treatments. This study examines the effectiveness and safety of early combination therapy with trimetazidine and either a β-blocker or a calcium channel blocker (CCB) among Egyptian patients with stable angina. **Methods:** This sub-analysis of the Egyptian cohort from the COMBINE Angina study included patients with stable angina (CCS class II–III) who remained symptomatic after 2–4 weeks of β-blocker or CCB monotherapy. Patients received trimetazidine 35 mg modified-release twice daily as an add-on treatment for 4 months. The primary outcome was the Seattle Angina Questionnaire (SAQ-7) summary score, with secondary outcomes including SAQ-7 domain scores, CCS class, frequency of angina attacks, nitrate use, treatment satisfaction, adherence, and safety. **Results:** In the global COMBINE study involving 578 patients, the Egyptian cohort had 138 participants with an average age of 58.3 years. The SAQ-7 summary score improved from 42.2 at baseline to 76.8 at month 4 (mean change 34.6 points, 95% CI 31.3 to 38.0; *p* < 0.0001). All 138 patients contributed data at every visit for the SAQ-7 summary score. Patients in CCS class I increased from 0% to 56.5%, weekly angina attacks fell from 5.0 to 1.7 (*p* < 0.0001), and short-acting nitrate use decreased from 2.9 to 1.0 doses per week (*p* < 0.0001). Patient satisfaction was about 75%, and physician satisfaction reached 83% by month 4. Medication adherence also improved (MARS-5 score: 20.8 to 22.7), and two non-serious, treatment-related adverse events (nausea and somnolence) were reported in a single patient (0.7%), with no serious or fatal events. **Conclusions:** In Egyptian patients with stable angina, implementing an early combination strategy incorporating trimetazidine with first-line β-blocker or CCB therapy was associated with clinically meaningful improvements in angina symptoms, functional status, and quality of life, with good tolerability and high satisfaction. As the study was observational and single-arm, these findings are provisional and require confirmation in further long-term controlled studies.

## 1. Introduction

Stable angina is the key manifestation of chronic ischemia to the coronary circulation [1]. It mostly relies on atherosclerosis to result in chest pain or dyspnea, which is usually precipitated by exertion or stress [2].

Epidemiologically, the high burden of angina is represented by the global estimation of 112 million cases [3], and Egypt ranks among the countries with the highest burden of angina, according to global ischemic heart disease (IHD) analysis across 204 countries [4].

Angina symptoms are frequently disabling, leading to a reduced quality of life for many patients and imposing a burden on healthcare costs [5,6]. In Egypt, angina pectoris is stigmatized as a leading cause of mortality nationwide, which can be attributed to the high prevalence of cardiovascular risk factors, such as diabetes mellitus (DM), hypertension (HTN), dyslipidemia, and smoking [7,8].

In everyday clinical practice, angina is primarily managed through lifestyle modification, abandoning bad habits like smoking and sedentariness [9]. In addition, pharmacological therapy is prescribed to reduce myocardial oxygen demand, including β-blockers and calcium channel blockers (CCBs) as first-line agents [10]. However, symptom alleviation cannot be achieved in all cases, particularly in those with multiple comorbidities or persistent ischemia [11]. Subsequently, anginal attacks are still a major challenge in the lives of these patients despite following the treatment protocol, highlighting the need for a tailored and more advanced therapy [12].

Trimetazidine is an agent that can complement the hemodynamic mechanisms of standard anginal drugs, which mainly aim to minimize the demand for oxygen [13]. It acts by inhibiting 3-ketoacyl-CoA thiolase (3-KAT), shifting myocardial cell metabolism from fatty acid oxidation, a more oxygen-consuming energy pathway, to a less oxygen-consuming pathway involving glucose [14]. Additionally, this contributes to the decrease in the generation of free radicals and acidosis, minimizing the impact of ischemia and maintaining contractile function [13].

Several reports have explored the combination of β-blockers or CCBs and trimetazidine, which showed promising results, including angina frequency reduction, exercise tolerance, and quality of life improvement [15,16,17,18]. The European Society of Cardiology (ESC) Guidelines acknowledge that myocardial ischemia is multifactorial in nature, and suggest that combining antianginal agents with complementary mechanisms of action may be beneficial in patients not adequately controlled by first-line treatment [19]. This was demonstrated in the prospective, observational COMBINE Angina study, which evaluated the effectiveness and safety of add-on trimetazidine in recently diagnosed patients with persistent angina despite first-line ß-blocker or CCB therapy [20]. COMBINE Angina showed that combining the metabolic effects of trimetazidine with the hemodynamic effects of a ß-blocker or CCB rapidly improved angina symptoms, physical functioning, and quality of life (QoL) and was well tolerated.

However, limited evidence remains regarding the impact of this combination in real-world settings, including the Egyptian population. This subgroup analysis of the COMBINE Angina study aimed to assess the effectiveness and safety of early trimetazidine combination therapy among Egyptian patients recently diagnosed with stable angina. Additionally, the evaluation focused on angina symptom control, physical limitation, and QoL improvement using the Seattle Angina Questionnaire-7 (SAQ-7). Therefore, the study will provide practical insight into optimizing antianginal management and improving patient outcomes in routine clinical practice.

## 2. Materials and Methods

### 2.1. Study Design

The Egyptian COMBINE Angina study is part of the international COMBINE Angina project [20], designed as a prospective, observational, single-arm, real-world study. The study was conducted at seven clinical sites across Egypt between 23 December 2023 and 9 December 2024, adhering to ethical principles of the Declaration of Helsinki, Good Pharmacoepidemiology Practices, and all applicable national regulations. Patients were asked to sign an informed consent form before enrollment. Additionally, ethical approval was obtained from the Institutional Review Board (IRB) of the Ministry of Health and Population/Centralized Directory for Research and Health Development, Egypt on 6 December 2023 (Approval No. 21-2023/33).

### 2.2. Study Population

Adult patients (≥18 years) were eligible if they had been recently diagnosed with stable angina pectoris (Canadian Cardiovascular Society [CCS] class II–III), including patients with vasospastic angina, which in COMBINE Angina was defined pragmatically as angina occurring at rest; no invasive or non-invasive provocation testing (for example, an acetylcholine provocation test) was required or performed, and the diagnosis rested on the treating physician’s clinical assessment. Additionally, the study included patients who reported ongoing angina symptoms despite 2–4 weeks of first-line treatment with a single hemodynamic agent, either a β-blocker or a CCB, in accordance with the 2019 ESC Guidelines for Chronic Coronary Syndromes. We evaluated patients using the SAQ-7, including only patients with a score ≤ 60 on the SAQ-7 angina frequency domain, which corresponds to daily or weekly angina. The diagnosis of stable angina and the decision to initiate trimetazidine were made exclusively by the treating physician according to clinical judgment and routine medical practice; objective documentation of coronary artery disease or of myocardial ischemia was not an entry requirement, consistent with the non-interventional design of the study.

On the other hand, patients were excluded if they refused to participate, were already enrolled in other clinical studies, or had been previously treated with trimetazidine. Additionally, patients with severe renal impairment or movement disorders such as Parkinson’s disease or any other trimetazidine contraindication were excluded. Also, pregnant patients or those who are planning for pregnancy were excluded, along with those who have been subjected to revascularization by coronary artery bypass grafting (CABG) or percutaneous coronary intervention (PCI) for either acute coronary syndrome (ACS) or stable IHD. Moreover, we excluded patients with a history of various cardiovascular conditions that require pharmacological treatment, including ACS, either acute or previous, heart failure (HF), significant valvular disease, and cardiomyopathy.

### 2.3. Treatment and Patient Follow-Up

The study encompassed four visits across four months of follow-up, with the treating physician initiating trimetazidine as an add-on therapy for eligible patients with persistent angina despite first-line β-blocker or CCB therapy at baseline. In the Egyptian cohort, all 138 patients (100%) received trimetazidine 35 mg modified-release tablets twice daily (70 mg/day); no other dose or formulation was used, and the first dose was taken on the day of the inclusion visit. Additionally, patients’ demographics, clinical characteristics and medical histories were obtained at visit 1. The remaining visits were scheduled at two weeks, one month and four months after trimetazidine initiation, and at all four visits patients were evaluated for angina severity using both the CCS classification and the SAQ-7, while physicians recorded the number of angina attacks and of short-acting nitrate doses used during the previous week [21,22].

Medication adherence was evaluated using the 5-item Medication Adherence Report Scale (MARS-5) at every visit, including visit 1 [23]. The visit 1 (baseline) MARS-5 assessment was completed before the first dose of trimetazidine and therefore refers to adherence to the pre-existing first-line antianginal therapy; the MARS-5 assessments at weeks 2, month 1 and month 4 refer to the trimetazidine-based combination. Treatment satisfaction was assessed from both patients and physicians on a 5-point Likert scale. Any change in antianginal therapy (addition of a new agent, dose modification, or permanent discontinuation), any change in other concomitant cardiovascular therapy, and any coronary revascularization procedure were recorded at each follow-up visit. Moreover, adverse events (AEs) were recorded throughout the study and coded using the Medical Dictionary for Regulatory Activities (MedDRA, version 27.1). Physicians documented their recommendations at the final visit, including whether they intended to continue the same antianginal regimen beyond study completion.

### 2.4. Outcome Measures

The primary endpoint was calculating the mean SAQ-7 summary score at 4 months, reflecting patient-reported angina symptoms frequency, physical limitation, and quality of life.

Secondary endpoints included evaluation of SAQ-7 summary (which represents an integration of the patient’s physical limitation, angina symptoms and quality of life) at 2 weeks and 1-month post-inclusion; shifts in CCS class over time; changes in angina attack frequency and nitrate consumption; patient and physician satisfaction with treatment; Medication adherence (MARS-5 score); and the incidence and severity of treatment-emergent AEs during follow-up.

### 2.5. Statistical Analysis

The Egyptian cohort contributed to the overall COMBINE Angina sample of 578 patients across five countries; no separate sample-size calculation was performed for this country-level sub-analysis, which is therefore exploratory and was not powered for hypothesis testing. Two populations were defined. The safety set comprised all 143 Egyptian patients who received at least one dose of trimetazidine. The analysis population comprised the 138 patients who met all eligibility criteria, received add-on trimetazidine and had an evaluable baseline and at least one evaluable post-baseline SAQ-7 assessment; five of the 143 treated patients were excluded on these grounds. All effectiveness analyses reported here are based on the 138-patient analysis population. Descriptive statistics were used to summarize patient demographics, baseline characteristics and clinical outcomes: mean and standard deviation (SD), median, first and third quartiles and range for continuous variables, and frequencies and percentages for categorical variables, with percentages calculated on the number of patients with a non-missing value. The distribution of the continuous variables and of their change scores was examined before parametric testing by inspecting histograms and normal quantile–quantile plots together with the agreement between the mean and the median and the degree of skewness; the change scores for the SAQ-7 summary and domain scores were approximately symmetric, and with 138 paired observations the paired *t*-test is robust to moderate departures from normality by the central limit theorem.

Changes from baseline in continuous outcomes (SAQ-7 summary and domain scores, number of angina attacks, short-acting nitrate use and MARS-5 score) were tested using paired *t*-tests, as pre-specified in the study protocol and statistical analysis plan, and are reported as the mean change with its 95% confidence interval (CI) and the standardized within-patient effect size (Cohen’s dz). The counts of angina attacks and of nitrate doses are right-skewed; medians and interquartile ranges are reported alongside the means. The distribution of patients across CCS classes at each visit was compared with baseline using the chi-square, Cochran–Mantel–Haenszel or Cochran’s Q test according to the distribution, as pre-specified in the statistical analysis plan; because these tests do not exploit the paired structure of the data, the within-patient CCS transitions (improvement, no change, worsening relative to baseline) were additionally tested with an exact binomial (paired sign) test. Analyses were performed on observed data at each time point, and no imputation for missing data was performed; the number of patients contributing data is reported for every outcome and visit. Because this is a single-arm sub-analysis, no adjustment for multiplicity was applied, and all *p*-values are descriptive. A mixed-effects model for repeated measures was applied to the SAQ-7 summary score in the parent international study but was not repeated for the Egyptian subgroup. Statistical analyses were conducted using SAS Viya 3.0 (SAS Institute, Cary, NC, USA).

### 2.6. Ethics Declaration

This study was conducted in accordance with the ethical principles of the Declaration of Helsinki, Good Pharmacoepidemiology Practices, and all applicable national regulations. Ethical approval was obtained from the Institutional Review Board (IRB) of the Ministry of Health and Population/Centralized Directory for Research and Health Development, Egypt (Approval No. 21-2023/33).

## 3. Results

### 3.1. Baseline Characteristics

A total of 143 Egyptian patients received at least one dose of add-on trimetazidine and constituted the safety set; 138 of them met all eligibility criteria and had evaluable baseline and post-baseline SAQ-7 data and formed the analysis population described below. All 138 patients attended the month-4 visit, and no patient withdrew consent, was lost to follow-up or discontinued trimetazidine during the four months of follow-up. Our cohort was composed of 138 patients recently diagnosed with stable angina, with a mean (SD) age of 58.3 (8.3) years (range 38–80) and a male predominance (57.2%). Patients were largely overweight or obese (87.0%), with a mean (SD) BMI of 30.1 (5.1) kg/m^2^. Nearly half of the participants had never smoked (49.3%), while current smokers accounted for 37.7%. Hypertension was the most common comorbidity (55.1%), followed by dyslipidemia (42.8%) and diabetes mellitus (31.2%). The mean (SD) time since the diagnosis of angina was 1.5 (1.3) months.

Angina was effort-related in most patients, being triggered by a predictable exercise threshold in 102 patients (73.9%) and by an unpredictable threshold in 34 (24.6%), while 2 patients (1.4%) were classified as having resting (vasospastic) angina; 21 patients (15.2%) reported at least one attack occurring at rest during the week before the inclusion visit. Ischemia had been documented on a non-invasive test in 40 patients (29.0%) and was confirmed by that test in 38 of them. Patients reported an average of 5 angina attacks per week, and 73.9% had CCS class II and 26.1% CCS class III angina. The mean (SD) SAQ-7 summary score was 42.2 (13.6). By design, every patient was receiving a single first-line hemodynamic agent when trimetazidine was added: 113 patients (81.9%) were taking a β-blocker and 24 (17.4%) a CCB. No patient was receiving a β-blocker and a CCB concurrently, and one patient (0.7%) was recorded as receiving neither at the time of trimetazidine initiation. A resting heart rate above 70 bpm was present in 111 patients (80.4%). Baseline characteristics are shown in detail in Table 1.

### 3.2. Evolution of SAQ-7 Scores over Time

**Figure 1:**
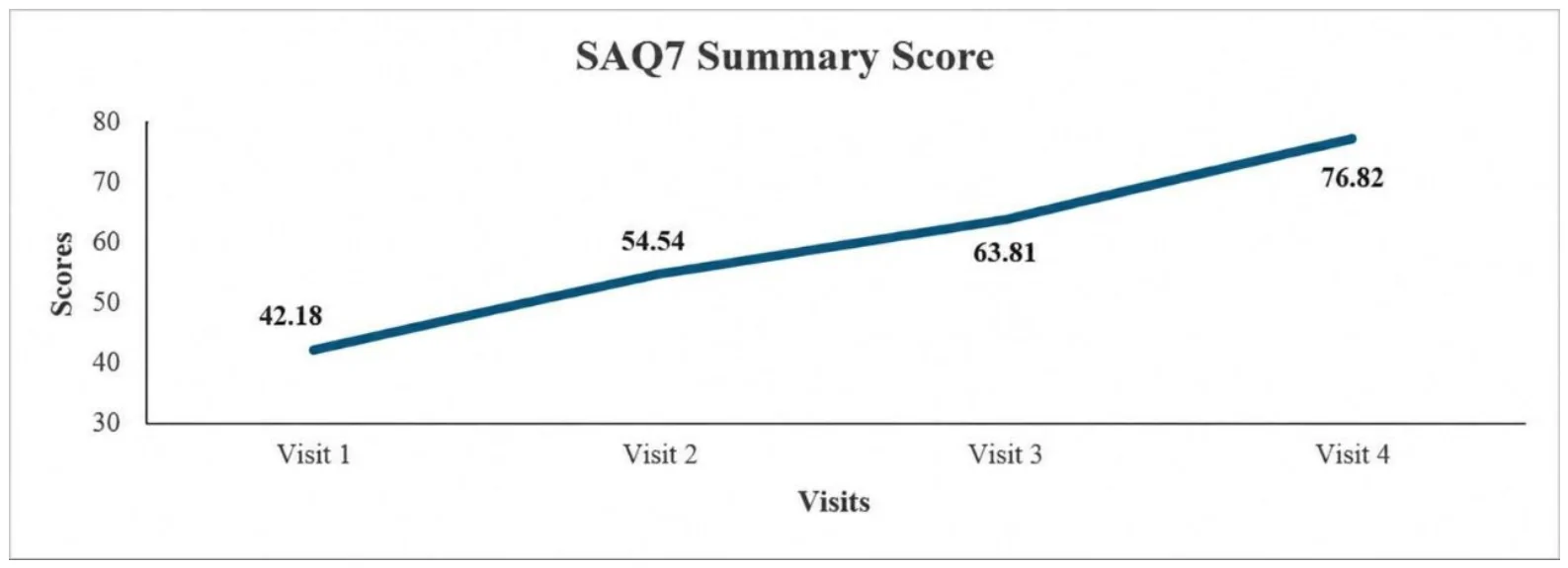
Evolution of SAQ-7 summary score over time.

Table 2 and Figure 1 show significant improvement across all SAQ-7 domains over the 4-month follow-up period, with the mean (SD) SAQ-7 summary score increasing from 42.2 (13.6) at baseline to 76.8 (17.2) at month 4. The mean within-patient increase at month 4 was 34.6 points (95% CI 31.3 to 38.0; *p* < 0.0001), corresponding to a large standardized effect size (Cohen’s dz = 1.73) and to roughly seven times the 5-point change that is generally regarded as the smallest clinically important difference on the SAQ-7 summary score. Improvement was already evident at two weeks (mean change 12.4 points, 95% CI 9.7 to 15.0; *p* < 0.0001) and increased at one month (21.6 points, 95% CI 18.4 to 24.8; *p* < 0.0001). Across domains, physical limitation, angina frequency and quality of life scores also rose progressively from 46.2 (18.2), 45.1 (14.9) and 35.0 (18.0) at baseline to 79.8 (18.6), 76.9 (20.5) and 73.7 (18.9) at month 4, respectively (all *p* < 0.0001); the corresponding mean changes at month 4 were 34.0 points (95% CI 30.0 to 38.0), 31.7 points (95% CI 28.4 to 35.1) and 38.7 points (95% CI 34.4 to 43.0), with effect sizes of 1.47, 1.58 and 1.52 (Table 3).

The SAQ-7 angina frequency, quality of life and summary scores were available for all 138 patients at every visit; the physical limitation score could not be computed for between one and three patients per visit because two or more items were answered “limited for other reasons or did not do the activity”, and the number of evaluable patients is given for each visit in Table 2. Notably, the proportion of patients reporting excellent physical status increased from 11.7% at baseline to 80.7% at month 4. In addition, patients experiencing daily or weekly angina declined from 100% at baseline to 29.7% at month 4, and 34 patients (24.6%) reported no angina at all at month 4.

### 3.3. Angina Grading and Symptom Evolution

More than half of the patients (78/138, 56.5%) had shifted from moderate-to-severe angina (CCS class II–III) at baseline into CCS class I at month 4, with 55 patients (39.9%) in class II and only 5 (3.6%) remaining in class III; no patient was in class IV. Relative to baseline, CCS class had improved in 91 patients (65.9%), was unchanged in 46 (33.3%) and had worsened in 1 (0.7%) at month 4; the corresponding figures were 48 (34.8%), 86 (62.3%) and 4 (2.9%) at week 2, and 67 (48.9%), 65 (47.4%) and 5 (3.6%) at month 1 (*n* = 137 at month 1, one value missing). Improvement significantly outweighed worsening at every visit (exact binomial test on the within-patient transitions, all *p* < 0.0001); the pre-specified comparison of the CCS class distribution against baseline gave *p* < 0.0001 at week 2, *p* = 0.1587 at month 1, and *p* = 0.0205 at month 4.

**Figure 2:**
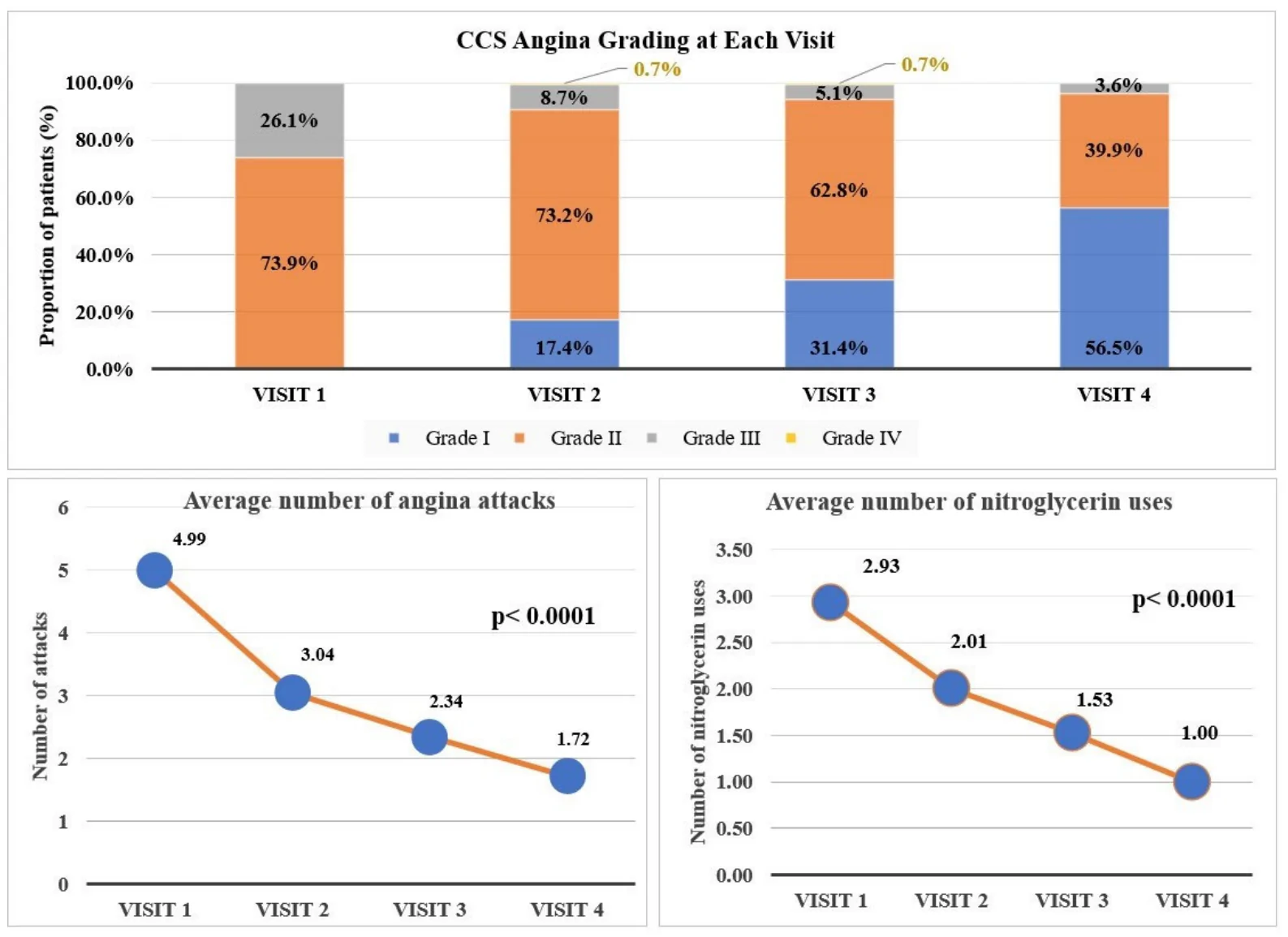
Changes in Angina Grading, Angina Attacks, and Nitroglycerin Use Across Follow-up Visits.

Additionally, a marked reduction in the frequency of angina attacks was noticed, from a mean (SD) of 4.99 (5.01) attacks/week at baseline to 1.72 (3.26)/week at month 4, a mean reduction of 3.33 attacks/week (95% CI 2.64 to 4.02; *p* < 0.0001), i.e., a 66% relative reduction. The mean (SD) short-acting nitrate use was also reduced from 2.93 (4.32) to 1.00 (2.25) doses/week (*p* = 0.0030 at week 2, *p* < 0.0001 at months 1 and 4). Data on angina attacks were available for 138, 138, 134, and 136 patients, and data on nitrate use for 136, 137, 132, and 136 patients at baseline, week 2, month 1, and month 4, respectively. Attacks occurring at rest fell from 21 patients (15.2%) at baseline to 1 (0.7%) at month 4, Figure 2.

### 3.4. Changes in Antianginal and Concomitant Therapy During Follow-Up

Changes to background therapy during the four months of follow-up were infrequent. At least one change in the antianginal treatment since the previous visit was recorded in 23 patients (16.7%) at week 2, 14 (10.1%) at month 1 and 3 (2.2%) at month 4; these changes consisted of a dose increase in an agent already being taken in 16, 12 and 0 patients, respectively, and of the addition of a new antianginal agent in 7, 2 and 3 patients, respectively. No antianginal treatment was permanently discontinued. A need for antianginal treatment upgrading because of an estimated failure of the combination under evaluation was recorded in 18 patients (13.0%) at week 2 and 13 (9.4%) at month 1, and in almost all of these the upgrade was an increase in the dose of an ongoing agent (18/18 and 12/13, respectively) rather than the addition of a new drug. A change in other, non-antianginal concomitant cardiovascular treatment was recorded in 6 patients (4.3%) at week 2, 5 (3.6%) at month 1, and none at month 4. No patient underwent coronary revascularization (percutaneous coronary intervention or coronary artery bypass grafting) during the study, and no patient had both a revascularization and an antianginal upgrade.

Cumulatively over the four months, 111 patients (80.4%) had neither a revascularization nor any antianginal treatment upgrade, 23 (16.7%) had one such event, and 4 (2.9%) had two. Thus, for approximately four out of five patients the antianginal regimen at month 4 was the β-blocker or CCB plus trimetazidine that had been started at baseline, unchanged in agent and in dose; nevertheless, the dose adjustments and additions that did occur in the remaining patients may have contributed to the improvement observed and cannot be separated from the effect of trimetazidine in a single-arm design.

### 3.5. Treatment Satisfaction, Adherence, and Safety

At week 2, just over half of the patients who completed the assessment (72/136, 52.9%) were satisfied or very satisfied with treatment; this proportion rose to 75.7% (103/136) by month 4. Physician satisfaction also increased progressively across all three domains, reaching 83.7% for effectiveness, 88.1% for tolerance and 85.2% for adherence at month 4 (*n* = 135 evaluations). The satisfaction assessment was not performed in 2, 7, and 2–3 patients at week 2, month 1, and month 4, respectively, and percentages are calculated on the patients assessed. Most physicians (135/138, 97.8%) recommended continuing the current therapy; a change in antianginal treatment, a coronary angiogram, and a revascularization procedure were recommended for one patient each (0.7% each). Additionally, patient-reported adherence improved, with MARS-5 scores rising from 20.81 (3.47) at baseline (*n* = 138) to 22.71 (2.41) at month 4 (*n* = 136).

With respect to safety, a total of two treatment-emergent adverse events were reported in one patient of the 138-patient analysis population (0.7%) and in one patient of the 143-patient safety set (0.7%): nausea and somnolence, both non-serious, both assessed by the investigator as related to trimetazidine, and both resolved within three days without treatment discontinuation. No serious, fatal or treatment-limiting adverse event occurred, and no patient discontinued trimetazidine because of an adverse event.

Given the size of the cohort and the four-month observation period, this cohort can exclude only a treatment-related adverse-event rate above approximately 4% (the exact 95% confidence interval around 1 event in 138 patients runs from 0.02% to 3.97%), and the safety observations should therefore be regarded as descriptive and reassuring rather than definitive.

## 4. Discussion

Myocardial ischemia is defined as a mismatch between myocardial oxygen supply and demand, which may manifest clinically as angina [21]. Both conditions may stem from multiple pathophysiological mechanisms, either separately or combined, including obstructive epicardial coronary disease, epicardial vasospasm, endothelial dysfunction, and coronary microvascular dysfunction [22]. This reflects the heterogenous basis of angina, inviting an individualized, mechanism-informed approach to symptom management rather than reliance on a single pharmacological pathway [23,24].

The contemporary ESC Guidelines for the management of chronic coronary syndromes support this concept [19]. They highlight the need for combination treatments in many patients to achieve adequate symptom control, rather than confining care to selective monotherapy. They further elaborate that the selection of antianginal therapy should be individualized according to the patient’s hemodynamic profile, comorbidities, concomitant medications, tolerability, preferences, and the underlying pathophysiological basis of myocardial ischemia [19].

Within this framework, β-blockers and/or CCBs remain important initial antianginal therapies, while other agents, including trimetazidine, may be added to a β-blocker and/or CCB or used as part of an initial combination strategy in appropriately selected patients [25,26]. Aligning with this evidence, we investigated the real-world impact of adding trimetazidine (a metabolic agent) on top of the standard antianginal therapy with β-blockers or CCBs (hemodynamic agents) in Egyptian patients recently diagnosed with symptomatic stable angina. We aimed to evaluate the clinical effectiveness, safety, adherence, and health-status consequences of this early combination therapy of trimetazidine on top of β-blockers or CCBs in routine clinical practice rather than to isolate the contribution of either component.

Following implementation of the early combination strategy, substantial improvements were observed across patient-reported health status, functional limitation, angina symptoms, and treatment adherence. SAQ-7 scores increased significantly as early as 2 weeks (*p* < 0.0001), with further improvement by month four. The mean SAQ-7 summary score increased from 42.2 at baseline to 76.8 at month four, a mean within-patient gain of 34.6 points (95% CI 31.3 to 38.0). The magnitude of this change is not only statistically significant but also clinically meaningful. It is about seven times the 5-point threshold usually taken as the smallest clinically important difference on the SAQ-7 and corresponds to a large standardized effect size (Cohen’s dz = 1.73) [27]. Additionally, it was accompanied by a shift in health status, as evidenced by the rise in the “excellent” category from none at baseline to 62.3% at month 4, indicating a marked improvement in patient-perceived health status.

These findings are also clinically relevant when considered alongside the broader spectrum of angina mechanisms. The absence of systematic objective documentation of obstructive coronary artery disease or inducible ischaemia in all participants reflects the clinical basis on which symptomatic stable angina was identified in this observational cohort. Contemporary evidence recognizes that angina/ischemia with non-obstructive coronary arteries (ANOCA/INOCA) is common among patients evaluated for anginal symptoms [28,29]. Therefore, the presence and severity of anginal symptoms do not necessarily reflect the degree of epicardial coronary obstruction alone [30]. ANOCA may be identified in up to 70% of patients investigated for undergoing invasive coronary angiography for angina [31].

Consistent with this perspective, a large contemporary cohort of 21,132 patients with ANOCA demonstrated that this population is not simply characterized by an absence of clinically meaningful cardiovascular risk [32]. They found 15-year risks of myocardial infarction and ischaemic stroke of 3.5% and 3.7%, respectively. Thus, the symptom-based clinical population represented in the present study is relevant to the heterogeneous spectrum of symptomatic chronic coronary syndromes, although objective assessment remains important when clinically indicated.

Similarly, the proportion of patients in the CCS classification shifted from 73.9% and 26.1% for class II and class III at the baseline visit to 56.5%, 39.9% and 3.6% for class I, class II and class III at month 4, respectively. This indicates a transition of anginal severity into milder forms, as reflected by the increase in the proportion of patients in CCS class I from 0% to 56.5% and by the fact that CCS class improved in 65.9% of patients while it worsened in a single patient. Furthermore, the reduction in symptom burden complements these findings. We observed a decrease in the frequency of angina attacks from an average of five per week at baseline to fewer than two per week at month 4, and a decline of approximately two-thirds in short-acting nitrate use during the week before each visit (mean 2.93 to 1.00 doses/week).

Taken together, the parallel improvements in health status, angina class, symptom frequency, and rescue medication use suggest clinically meaningful improvement following introduction of the early combination strategy. The observed pattern is consistent with the rationale for combining complementary antianginal mechanisms. β-blockers and CCBs provide hemodynamic modulation, whereas trimetazidine acts through a metabolic mechanism that improves myocardial energy efficiency under ischaemic conditions [33]. The clinical rationale for this combination is therefore not dependent on assigning the observed changes to a single pharmacological pathway, but rather on addressing myocardial ischaemia through complementary mechanisms within an individualized treatment strategy.

Beyond confirming the overall COMBINE Angina result in a single country, this Egyptian analysis describes a patient profile and treatment response that differ appreciably from the pooled international cohort. Egypt contributed the third-largest national sample, comprising 138 of 578 patients (23.9%), after India and Brazil. Compared with the pooled population, Egyptian patients were more often male (57.2% vs. 50.0%), more obese (mean BMI 30.1 vs. 28.6 kg/m^2^) and more than twice as likely to be current smokers (37.7% vs. 17.3%), yet had less hypertension (55.1% vs. 73.5%) and less dyslipidemia (42.8% vs. 61.9%) recorded at baseline, and presented with milder angina by CCS class (class II in 73.9% vs. 58.4%) but a higher consumption of short-acting nitrates (2.93 vs. 2.3 doses/week). All Egyptian patients received trimetazidine 35 mg twice daily, whereas 30.6% of the international cohort received the 80 mg once-daily formulation, and no Egyptian patient was receiving a β-blocker and a CCB together, compared with 6.9% internationally. The improvement observed in Egypt, although large, was more modest than in the pooled analysis: the SAQ-7 summary score rose by 34.6 points versus 39.2 points overall, and reached 76.8 at month 4 versus 78.4 overall, with 56.5% of Egyptian patients in CCS class I at month 4 versus 63.6% internationally.

The month-4 SAQ-7 summary score differed significantly between the five participating countries (Brazil 80.1, Egypt 76.8, India 72.7, Malaysia 70.9, Romania 85.5; *p* < 0.0001), which indicates that the pooled estimate conceals real country-level heterogeneity and supports the value of reporting national cohorts separately. The most striking national difference concerned adherence: the baseline MARS-5 score was 20.81 in Egypt against 23.2 internationally and remained lower at month 4 (22.71 vs. 24.1), identifying suboptimal adherence as a more prominent problem in this setting and a plausible target for intervention. Conversely, reported treatment-emergent adverse events were less frequent in Egypt (0.7%) than in the pooled cohort (3.3%), a difference that may reflect genuine tolerability but is at least as likely to reflect differences in adverse-event ascertainment and reporting between sites, and should not be over-interpreted.

Moreover, we assessed treatment satisfaction, tolerance and adherence, and found that more than 80% of treating physicians and approximately 75% of patients were either satisfied or very satisfied at month 4. These findings were further supported by the mean MARS-5 adherence score, which increased from 20.81 (3.47) at baseline to 22.71 (2.41) at month 4. Notably, physicians recommended continuing the same antianginal regimen for 135 of the 138 patients (97.8%), reflecting confidence in the practical value of this combination.

As per the ARCA Registry, a large observational prospective study, a stepwise guideline-guided medical treatment is superior to revascularization for treating stable angina [34]. The COMBINE Angina study is among the prospective real-world studies evaluating the effectiveness and safety of adding trimetazidine to conventional antianginal therapy, with its distinctive focus on patients early in the course of recently diagnosed stable angina. The broader literature aligns with our study’s findings regarding the safety and antianginal qualities of trimetazidine in combination with conventional antianginal therapy [26,35,36].

For instance, the CHOICE-2 observational study investigated administering 35 mg twice daily of trimetazidine in addition to conventional angina therapies in Russian patients with various durations of angina and found a significant decrease in weekly angina frequency (from 3.75 to 0.67 attacks) in a subset of patients with recently diagnosed stable angina (<1 year). They also agree with our study in concluding that the beneficial early combination of trimetazidine with anti-ischemic agents, as observed in their two-week findings, and the six-month maintenance [16]. This suggests that the symptomatic improvements associated with early combination of trimetazidine and β-blockers and/or CCB may persist beyond the four-month follow-up of our study.

A post hoc analysis of the CHOICE-2 study further supports the effectiveness of trimetazidine 35 mg twice daily when added to bisoprolol-based therapy in symptomatic patients with stable angina. They also reported a decrease in weekly angina episodes from 6.2 at baseline to 4.4 at 2 weeks and 1.5 at 6 months, alongside a reduction in short-acting nitrate use from 5.5 to 1.0 doses per week [37]. The proportion of angina-free patients increased from 5.4% at baseline to 33.9% at 6 months, while the proportion of patients with CCS class 1 angina increased and those with class 3 angina decreased, indicating sustained improvement in angina severity and functional status. Similarly, the ODA trial concurred with the same finding with 80 mg once daily trimetazidine, emphasizing great improvement in patients’ quality of life and a reduction in angina attacks and nitrate use [15]. These two studies confirm that the therapeutic effect of trimetazidine combined with conventional therapy is present earlier in the disease course, in addition to the advanced refractory cases.

The MODUS VIVENDI study further reinforced our findings across 1939 patients with moderate and severe stable angina who have been treated with bisoprolol [38]. A total of 250 healthcare physicians following the guidelines protocol were responsible for monitoring the results of adding trimetazidine 80 mg daily. They reported substantial improvements following treatment with this combination over three visits across three months, finding a statistically significant reduction in weekly anginal attacks (6.2 to 1.6 attacks at Visit 3), along with the consumption of short-acting nitrates (4.9 at Visit 1 to 1.1 at Visit 3). Additionally, a marked improvement in quality of life was documented, rising from 2.6% with no problems at Visit 1 to 44.0% at Visit 3, as measured by the EQ-5D-3L tool. Also, medication adherence was observed in their study, which progressively increased from 29.9% at baseline to 41.7% at Visit 2 and 53.8% at Visit 3.

Moreover, our findings are broadly consistent with those of the V-GOOD observational study conducted in Brazil, which evaluated trimetazidine modified-release 80 mg once daily in 1026 patients with chronic coronary syndrome [39]. They revealed early and sustained reductions in angina burden following the addition of trimetazidine to background antianginal therapy, noting a decrease in anginal attacks from 3.1 to 1.0 attacks/week at 1 month and to 0.7 attacks/week at 3 months. Additionally, they found a reduction in the use of short-acting nitrate from 2.6 to 0.7 and 0.6 doses/week. These findings are directionally consistent with our study, which also found a decrease in anginal attacks from 4.99 to 1.72 attacks/week by month 4 and short-acting nitrate use from 2.93 to 1.00 doses/week, despite the different population and treatment regimen (Egyptians with recently diagnosed angina receiving trimetazidine 35 mg twice daily vs. Brazilians with chronic coronary syndrome receiving 80 mg once-daily).

Regarding safety, the combination was also well tolerated, with two non-serious, treatment-related adverse events (nausea and somnolence) reported in a single patient (0.7%), both of which resolved within three days without treatment discontinuation, and with no serious or fatal events. This is consistent with the parent international study, in which treatment-related adverse events were reported in 4 of the 607 patients (0.7%) of the safety population. In V-GOOD, adverse events were reported in 6.1% of patients and adverse drug reactions in 1.8%, with dizziness and nausea among the most frequently reported reactions; no deaths were considered related to trimetazidine [39]. The lower event rate observed in the Egyptian cohort may reflect differences in population characteristics, duration of follow-up, event ascertainment, and reporting practices rather than a true difference in tolerability. This further supports the observation that adding trimetazidine to conventional antianginal therapy is generally well tolerated and associated with improvement in symptomatic burden in routine clinical practice [36,40]. However, a cohort of 138 patients followed for four months cannot exclude uncommon adverse effects, and these observations should be interpreted as supportive rather than as definitive evidence of safety.

While our study and the aforementioned real-world investigations provide valuable insights into the short-to-medium-term outcomes following the addition of trimetazidine, a meta-analysis of 13 randomized controlled trials involving 1628 patients provides complementary higher-level evidence for the incremental efficacy of trimetazidine when added to conventional antianginal therapy [36]. Although the populations and treatment regimens across the included trials were heterogeneous and were not restricted to recently diagnosed stable angina, the analysis demonstrated that adding trimetazidine significantly reduced weekly angina attacks and nitroglycerin use and improved exercise duration, total work, and time to 1-mm ST-segment depression compared with conventional antianginal therapy alone. Importantly, treatment duration was not a significant moderator of these outcomes, with no significant differences between studies evaluating treatment for up to 8 weeks and those evaluating treatment for more than 12 weeks. Together with the six-month findings from CHOICE-2, these data provide supportive evidence that the symptomatic benefits observed after adding trimetazidine may persist beyond the four-month follow-up of the present study.

Despite the insights provided by the Egyptian COMBINE Angina study, several limitations should be acknowledged. First, this was an observational, single-arm study without a parallel control group. Therefore, the observed changes should be interpreted as the clinical course following implementation of the early combination therapy of trimetazidine on top of β-blocker or CCB rather than as evidence from a randomized comparison. Other factors may also have influenced the observed changes, including regression to the mean, the natural course of recently diagnosed angina, and increased clinical contact during follow-up. Second, recall, expectation, and observer biases may have arisen from the use of patient- and physician-reported outcomes assessed without blinding, including the SAQ-7, CCS class, angina frequency, nitrate use, and MARS-5. Third, selection by treating physicians and the relatively young mean age of the cohort (58.3 years) may limit generalizability to other patient populations. Fourth, the Egyptian subgroup was not separately powered for inferential analyses; country-specific comparisons and *p*-values were therefore descriptive and were not adjusted for multiplicity. The sample size also limits the ability to evaluate uncommon adverse events. Finally, the four-month follow-up may not capture the long-term effects of the combination strategy on adherence and symptom control.

## 5. Conclusions

In conclusion, in Egyptian patients recently diagnosed with stable angina who remained symptomatic on a first-line β-blocker or CCB, implementation of an early combination therapy incorporating trimetazidine on top of a β-blocker or CCB was associated with clinically meaningful improvements in patient-reported angina symptoms, physical function and quality of life, with fewer angina attacks, less short-acting nitrate use, a shift towards milder CCS classes, improved adherence, and good tolerability over four months. These findings provide prospective observational evidence on the clinical course following early use of complementary hemodynamic and metabolic antianginal therapy in this population. They are also consistent with the contemporary guideline-supported role of individualized combination therapy for symptom control. Longer-term comparative studies are warranted to evaluate the durability and comparative effectiveness of this treatment strategy.

## Figures and Tables

**Table 1:** Baseline Demographic, Clinical, and Treatment Characteristics of the Egyptian Study Population (*n* = 138).

Baseline Characteristics (Visit 1)	Value
**Age (years)**	
Mean (SD)	58.3 (8.3)
**Male sex, *n* (%)**	79 (57.2)
**BMI (kg/m^2^)**	
Mean (SD)	30.1 (5.1)
**Categories, *n* (%)**	
Obese (≥30)	61 (44.2)
Overweight (25 to <30)	59 (42.8)
Normal weight (18.5 to <25)	17 (12.3)
Underweight (<18.5)	1 (0.7)
**Smoking status, *n* (%)**	
Current smoker	52 (37.7)
Former smoker	15 (10.9)
Never smoked	68 (49.3)
Unknown	3 (2.2)
**Comorbidities, *n* (%)**	
HTN	76 (55.1)
DM	43 (31.2)
Dyslipidemia	59 (42.8)
**Angina History**	
Number of angina attacks in the previous week, mean (SD)	4.99 (5.01)
Number of short-acting nitrates used in the previous week, mean (SD)	2.9 (4.3)
Time since angina diagnosis (months), mean (SD)	1.5 (1.3)
Type of angina, ***n***(%) *	
Effort angina, predictable threshold	102 (73.9)
Effort angina, unpredictable threshold	34 (24.6)
Resting (vasospastic) angina	2 (1.4)
Angina attacks occurring at rest in the previous week, ***n*** (%)	21 (15.2)
Documented ischemia on non-invasive testing, ***n*** (%)	40 (29.0)
Elevated resting heart rate (>70 bpm), ***n*** (%)	111 (80.4)
**CCS Classification, *n* (%)**	
CCS Class 2	102 (73.9)
CCS Class 3	36 (26.1)
**SAQ-7 Summary Score**	
Mean (SD)	42.2 (13.6)
**Health status based on SAQ-7 summary score, *n* (%)**	
Poor (score 0–24)	14 (10.1)
Fair (score 25–49)	87 (63.0)
Good (score 50–74)	37 (26.8)
Excellent (score 75–100)	0 (0.0)
Concomitant Antianginal Treatment at Baseline, *n* (%) *	
β-blocker	113 (81.9)
CCB	24 (17.4)
β-blocker and CCB concurrently	0 (0.0)
Neither a β-blocker nor a CCB	1 (0.7)
Short-acting nitrates	4 (2.9)
Antiplatelet agent	56 (40.6)
Oral anticoagulant	1 (0.7)
ACEI	5 (3.6)
ARB	13 (9.4)
Lipid-lowering drug	91 (65.9)
Diuretic	6 (4.3)
Anti-diabetic treatment	43 (31.2)

SD: Standard deviation; BMI: Body mass index; CCS: Canadian Cardiovascular Society; SAQ-7: 7-item Seattle Angina Questionnaire; HTN: Hypertension; DM: Diabetes mellitus; CCB: Calcium channel blocker; ACEI: Angiotensin-converting enzyme inhibitor; ARB: Angiotensin II receptor blocker. * More than one answer possible. Percentages are calculated on the number of patients with a non-missing value. Every patient was receiving one first-line hemodynamic agent at the time trimetazidine was added: no patient received a β-blocker and a CCB concurrently, so the β-blocker and CCB rows are mutually exclusive. Documented ischemia was confirmed by the non-invasive examination in 38 of the 40 patients tested (ECG exercise test, *n* = 14; stress echocardiography, *n* = 4; PET/SPECT/CT, *n* = 4; other, *n* = 18); no invasive or pharmacological provocation testing was performed.

**Table 2:** Evolution of SAQ-7 Domain Scores Over Time (*n* = 138).

Measure	Baseline	Week 2	Month 1		Month 4
**SAQ-7 Physical Limitation Score**					
Mean (SD)	46.2 (18.2)	58.4 (21.1)	67.04 (21.03)		79.8 (18.6)
**Patients with an evaluable score, *n***		137	137	136	135
**Health status according to SAQ-7 Physical Limitation Score, *n* (%)**					
Poor (score 0–24)	6 (4.4)	4 (2.9)	0 (0.0)		0 (0.0)
Fair (score 25–49)	60 (43.8)	31 (22.6)	20 (14.7)		8 (5.9)
Good (score 50–74)	55 (40.1)	56 (40.9)	48 (35.3)		18 (13.3)
Excellent (score 75–100)	16 (11.7)	46 (33.6)	68 (50.0)		109 (80.7)
**SAQ-7 Angina Frequency Score**					
Mean (SD)	45.1 (14.9)	58.3 (18.1)	66.2 (19.1)		76.9 (20.5)
**Patients with an evaluable score, *n***		138	138	138	138
**Angina frequency according to SAQ-7 Angina Frequency Score, *n* (%)**					
Daily (score 0–30)	31 (22.5)	9 (6.5)	3 (2.2)		5 (3.6)
Weekly (score 31–60)	107 (77.5)	90 (65.2)	73 (52.9)		36 (26.1)
Monthly (score 61–99)	0 (0.0)	36 (26.1)	52 (37.7)		63 (45.7)
None (score 100)	0 (0.0)	3 (2.2)	10 (7.2)		34 (24.6)
**SAQ-7 Quality of Life Score**					
Mean (SD)	34.99 (18.04)	47.02 (21.9)	58.14 (24.0)		73.7 (18.9)
**Patients with an evaluable score, *n***		138	138	138	138
**Health status according to SAQ-7 Quality of Life Score, *n* (%)**					
Poor (score 0–24)	21 (15.2)	14 (10.1)	6 (4.3)		1 (0.7)
Fair (score 25–49)	80 (58.0)	43 (31.2)	28 (20.3)		6 (4.3)
Good (score 50–74)	28 (20.3)	54 (39.1)	53 (38.4)		34 (24.6)
Excellent (score 75–100)	9 (6.5)	27 (19.6)	51 (37.0)		97 (70.3)
**SAQ-7 Summary Score**					
Mean (SD)	42.2 (13.6)	54.5 (16.9)	63.8 (18.95)		76.8 (17.15)
**Patients with an evaluable score, *n***		138	138	138	138
**Health status according to the SAQ-7 Summary score**					
Poor (0–24)	14 (10.1)	5 (3.6)	1 (0.7)		1 (0.7)
Fair (25–49)	87 (63.0)	56 (40.6)	37 (26.8)		9 (6.5)
Good (50–74)	37 (26.8)	64 (46.4)	56 (40.6)		42 (30.4)
Excellent (75–100)	0 (0.0)	13 (9.4)	44 (31.9)		86 (62.3)

Data are observed values at each visit; no imputation was performed. All SAQ-7 domain and summary scores differed significantly from baseline at every visit (paired *t*-test, all *p* < 0.0001); the corresponding mean changes with 95% confidence intervals are given in Table 3. Higher SAQ-7 scores indicate better physical function, less frequent angina and better quality of life. Percentages are calculated on the number of patients with a non-missing score at that visit. Scores were missing for 1–3 patients at individual visits in the physical limitation domain only (an SAQ-7 physical limitation score is not computed when two or more items are answered “limited for other reasons/did not do the activity”); the angina frequency, quality of life and summary scores were available for all 138 patients at every visit. SAQ-7: 7-item Seattle Angina Questionnaire; SD: Standard deviation.

**Table 3:** Change from baseline in SAQ-7 scores and in angina burden at each visit, with 95% confidence intervals and standardized effect sizes (Egyptian cohort).

Outcome and Visit	*N*	Mean Change from Baseline (SD)	95% CI	Cohen’s dz
**SAQ-7 summary score**				
Week 2	138	12.36 (15.55)	9.74 to 14.98	0.79
Month 1	138	21.63 (19.07)	18.42 to 24.84	1.13
Month 4	138	34.64 (20.03)	31.27 to 38.01	1.73
**SAQ-7 physical limitation score**				
Week 2	136	12.22 (21.51)	8.57 to 15.87	0.57
Month 1	135	21.17 (23.59)	17.15 to 25.19	0.90
Month 4	134	33.99 (23.19)	30.03 to 37.95	1.47
**SAQ-7 angina frequency score**				
Week 2	138	13.12 (16.16)	10.40 to 15.84	0.81
Month 1	138	21.01 (18.88)	17.83 to 24.19	1.11
Month 4	138	31.74 (20.07)	28.36 to 35.12	1.58
**SAQ-7 quality of life score**				
Week 2	138	12.03 (20.14)	8.64 to 15.42	0.60
Month 1	138	23.15 (23.41)	19.21 to 27.09	0.99
Month 4	138	38.68 (25.45)	34.40 to 42.96	1.52
**Angina attacks in the previous week (/week)**				
Week 2	138	−1.96 (4.09)	−2.65 to −1.27	−0.48
Month 1	134	−2.75 (4.22)	−3.47 to −2.03	−0.65
Month 4	136	−3.33 (4.05)	−4.02 to −2.64	−0.82

All changes from baseline were significant at *p* < 0.0001 (paired *t*-test). Positive values denote improvement for the SAQ-7 scores (higher score = better health status) and negative values denote improvement for the number of angina attacks. Cohen’s dz is the mean change divided by the SD of the change (0.2 = small, 0.5 = medium, 0.8 = large). A change of ≥5 points on the SAQ-7 summary score is generally considered the smallest clinically important difference, ≥10 points a moderate and ≥20 points a large clinical improvement. *n* = number of patients with both a baseline and a visit value. CI: confidence interval; SD: standard deviation.

## Data Availability

The original contributions presented in this study are included in the article. Further inquiries can be directed to the corresponding author.

## Abbreviations

3-KAT	3-ketoacyl-CoA thiolase
ACEI	Angiotensin-converting enzyme inhibitor
ACS	Acute Coronary Syndrome
AEs	Adverse Events
ARB	Angiotensin II receptor blocker
BMI	Body mass index
BP	Blood Pressure
CABG	Coronary Artery Bypass Grafting
CCB	Calcium Channel Blocker
CCS	Canadian Cardiovascular Society
ESC	European Society of Cardiology
HF	Heart Failure
HR	Heart Rate
HTN	Hypertension
IRB	Institutional Review Board
MARS-5	Medication Adherence Report Scale
MedDRA	Medical Dictionary for Regulatory Activities
PCI	Percutaneous Coronary Intervention
QoL	Quality of Life
SAQ-7	Seattle Angina Questionnaire
SD	Standard Deviation
